# Thermostable protein-stabilized gold nanoclusters as a peroxidase mimic†

**DOI:** 10.1039/d3na00566f

**Published:** 2023-09-21

**Authors:** Özlem Akyüz, Maite Mißun, Rose Rosenberg, Martin Scheffner, Andreas Marx, Helmut Cölfen

**Affiliations:** a Physical Chemistry, Department of Chemistry, University of Konstanz 78457 Konstanz Germany helmut.coelfen@uni-konstanz.de; b Organic Chemistry/Cellular Chemistry, Department of Chemistry, University of Konstanz 78457 Konstanz Germany; c Department of Biology, University of Konstanz 78457 Konstanz Germany

## Abstract

Protein-stabilized gold nanoclusters (AuNCs) are fascinating nanostructures with exciting properties owing to their ultra-small sizes and functional shell. However, their applications under extreme conditions are still complicated, waiting for programmable solutions. Therefore, the design of a multi-functional protein stabilizer for specific purposes gains attention to improve the stability and functionality of AuNCs. Herein, we exploited the thermostability of genetically engineered KlenTaq DNA polymerase containing five cysteine residues (KTQ5C) to synthesize heat-stable AuNCs (AuNC@KTQ5C) and characterize optical, structural, and hydrodynamic properties. Besides their excellent photophysical properties, AuNC@KTQ5C also exhibit superior peroxidase-like (POD-like) catalytic activity following typical Michaelis–Menten kinetics together with a high affinity towards the POD substrate 2,2′-azino-bis(3-ethylbenzothiazoline-6-sulfonic acid)-diammonium salt (ABTS). Moreover, FTIR and relative catalytic activity analysis of AuNC@KTQ5C reveal that KTQ5C is resistant to changes in protein secondary structure while the AuNCs conserve 70–80% of their catalytic performance after heat treatments up to more than 80 °C. Our findings show that stabilizing AuNCs with thermostable KTQ5C not only preserves the advantages of protein-stabilized AuNCs but can also promote the resistance of AuNCs against aggregation due to protein denaturation under extreme reaction temperatures, protecting their fluorescent emission or catalytic activity.

## Abstract 2

By using a genetically modified thermostable protein (KTQ5C), we have synthesized protein-stabilized goldnanoclusters (AuNC@KTQ5C) with advantageous properties, such as heat stable fluorescent emission and heat resistant peroxidase-like activity.

## Graphical abstract

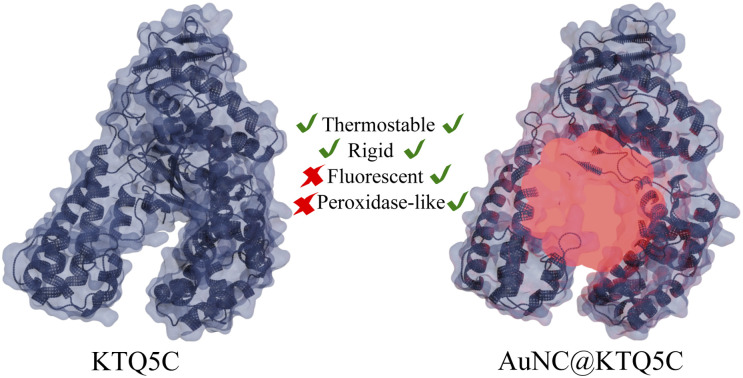

## Introduction

There has been an ongoing interest in the use of noble metal nanoclusters (NC) for a broad range of biomedical and catalytic applications, owing to their ultra-small sizes (<3 nm and comprised of a few to several hundreds of atoms), biocompatibility, and stable fluorescent emission.^1–3^ Particularly, protein-stabilized NCs have come into prominence over the last years and have been extensively utilized for bio-imaging,^4–6^ sensing,^7–9^ and bio-catalysis.^10,11^ Among the various NC, gold nanoclusters (AuNCs) stabilized in different proteins such as bovine serum albumin (BSA),^12–14^ ferritin,^15^ lysozyme,^16^ insulin,^17^ and fibrinogen^18^ have been widely reported to show outstanding optical and catalytic performances. These properties are heavily dependent on the structural integrity, size, and conformation of the stabilizers.^19,20^ Therefore, encapsulating the AuNC with a proper protein is crucial to get an efficient optical and catalytic response. It is known that AuNCs are utilized as enzyme mimics in several applications^21^ where the natural counterpart has lower activity, stability, and reusability.^22,23^ However, the utilization of natural proteins, previously mentioned, as stabilizing agents is limiting the catalytic performance and application fields of biocompatible AuNC with low denaturation temperatures. Thus, the combination of the versatile properties of AuNCs with a rigid protein could lead to a broader application area for these enzyme mimics.

The Klenow fragment of Thermus aquaticus (Taq) DNA polymerase I (in short KlenTaq) is a cysteine-free and well-known thermostable enzyme which imparts high stability at 98 °C for 40 min in polymerase chain reactions.^24^ KlenTaq is a truncated version of Taq DNA polymerase, lacking the N-terminal 280 amino acids. The enzyme catalyzes the polymerization of nucleotides into duplex DNA in 5′ → 3′ direction in the presence of magnesium but lacks the nuclease activity of Taq DNA polymerase. In addition, KlenTaq possesses a binding nanopocket enabling the accumulation of metal ions in a region where the saturation concentration can be readily increased.

**Fig. 1:**
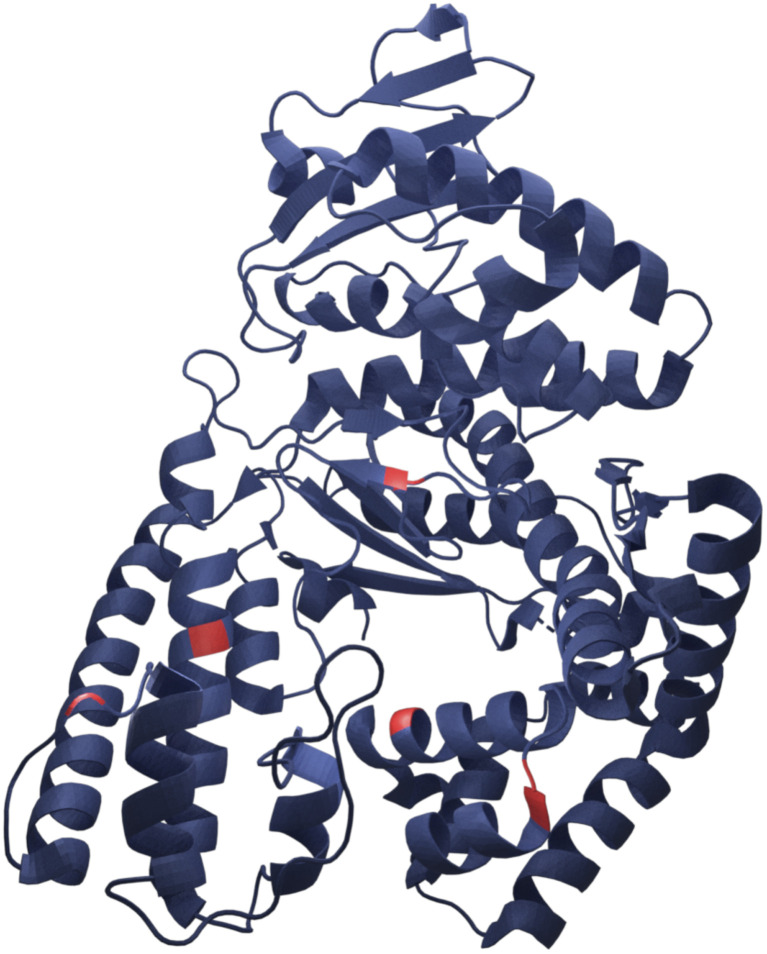
Representative crystal structure of KlenTaq showing the five mutation positions in red (RCSB Protein Data Bank, PDB ID 6Q4V).

Here we present successful nucleation and growth of AuNCs likely in the protein nanopocket^25^ of KlenTaq where five cysteine residues (N192C K249C S285C R370C S384C) were introduced (KTQ5C) (Fig. 1). Moreover, we investigated a potentially modulated performance of the designer thermostable KTQ5C stabilized catalytically active AuNCs^26^ (AuNC@KTQ5C) in terms of its thermal stability and catalytic activity. We characterized the synthesized AuNC@KTQ5C by UV-vis and fluorescence spectroscopy, high-resolution transmission electron microscopy (HR-TEM), analytical ultracentrifugation (AUC), and infrared spectroscopy (IR). The efficiency of the as-synthesized AuNC@KTQ5C as a peroxidase-mimicking (POD-mimicking) nanozyme under extreme temperatures was demonstrated by exploiting the enzymatic oxidation reaction of ABTS to the colored product ABTS˙^+^ in the presence of H_2_O_2_.^27^ Our AuNCs design was investigated for the biocompatibility, thermostability, and rigidity of the stabilizer KTQ5C to present its highly versatile usability.

## Experimental

### Materials

Hydrogen tetrachloroaurate trihydrate (HAuCl_4_·3H_2_O, ≥99.9%), bovine serum albumin (BSA), sodium hydroxide granules (NaOH), and horseradish peroxidase (HRP) were purchased from Sigma Aldrich. Hydrogen peroxide (H_2_O_2_) and 2,2′-azino-bis(3 ethylbenzothiazoline-6-sulfonic acid)-diammonium salt (ABTS) were obtained from Merck. Milli-Q water (resistivity 18.2 MΩ cm) was used in all experiments. The codon-optimized cDNA of KTQ5C (N192C K249C S285C R370C S384C) was ordered as a gene block from IDT.

### Expression and purification of KTQ5C

The cDNA encoding KTQ5C was inserted into pET21a and transformed into *E. coli* BL21(DE3). Cells were cultured in LB medium containing 100 mg L^−1^ carbenicillin at 37 °C. At OD600 = 0.6, 1 mM IPTG was added to induce protein expression. After 4 h, cells were harvested by centrifugation (4000 rpm, 10 min, 4 °C). Pellets were resuspended in lysis buffer (Tris–HCl pH 8.55 50 mM, MgCl_2_ 10 mM, (NH_4_)_2_SO_4_ 16 mM, Thesit 0.1% (v/v), Triton X-100 0.1% (v/v), dithiothreitol (DTT) 2 mM) and lysed for 2 h at 37 °C by addition of 0.5 mg mL^−1^ lysozyme and 0.2 mM PMSF. The lysate was incubated at 75 °C for 20 min to denature the heat-labile proteins and cleared by centrifugation (35 000 g, 1 h, 4 °C). Genomic DNA was precipitated by dropwise addition of 5% PEI (*M*_w_: 25 kDa) (100 μL) and incubation on ice for 30 min. Subsequently, suspensions were centrifuged (4000 rpm, 30 min, 4 °C) and purified *via* a 5 mL HiTrap Q HP (Cytiva) with a linear gradient from 0 to 2 M sodium chloride (buffer A: Tris–HCl pH 8.55 20 mM, EDTA 1 mM, DTT 2 mM buffer B: Tris–HCl pH 8.55 20 mM, EDTA 1 mM, DTT 2 mM, NaCl 2 M). Fractions containing the desired protein were analyzed *via* SDS-PAGE, pooled, and purified *via* size exclusion chromatography on a HiLoad 16/600 75 pg (Cytiva) using Tris–HCl pH 8.55 20 mM, EDTA 1 mM, NaCl 150 mM, DTT 2 mM. Pure fractions were confirmed by sodium dodecyl-sulfate polyacrylamide gel electrophoresis (SDS-PAGE) analysis, pooled, and concentrated using Amicon® Ultra (30 kDa MWCO). The concentration was determined by nanodrop (extinction coefficient: 70 360 M^−1^ cm^−1^). The protein was dialyzed against Milli-Q water at room temperature overnight to remove excess reducing agent and buffer, which could affect the nucleation and growth processes.

### Synthesis of AuNC@KTQ5C

KTQ5C stabilized AuNCs were synthesized using the reported method by Xie *et al.*^28^ First, optimization of the gold concentration (from 1.0 to 12 mM) with constant protein concentration (4 mg mL^−1^) was achieved by measuring the fluorescence intensity of the prepared AuNCs. The optimum concentrations of KTQ5C and HAuCl_4_ were 4 mg mL^−1^ and 2.0 mM, respectively, in a final volume of 0.6 mL. After short incubation of KTQ5C with HAuCl_4_ at 37 °C, 10 μL NaOH solution (1.0 M) was added to make the solution of pH 12 and further incubated at 37 °C overnight.

### Characterization of AuNC@KTQ5C

Varian Cary 50 UV-Vis Spectrophotometer and Cary Eclipse Fluorescence Spectrophotometer were used to record the absorption and fluorescence spectra, respectively. To investigate the size and size distribution of the AuNCs, a JEOL JEM-2200FS- High-Resolution Transmission Electron Microscope (HRTEM) was utilized. For this purpose, the sample was placed on a thin carbon-coated Cu grid and air-dried. The HRTEM images were analyzed by DigitalMicrograph (Gatan). Matrix-assisted laser desorption/ionization–time-of-flight (MALDI-TOF) was performed with a Bruker Microflex TOF mass spectrometer. Attenuated-total-reflection Fourier-transform infrared spectroscopy (ATR-FTIR) was employed to analyze the interaction of the protein with AuNCs and the secondary structural changes of the protein by deconvolution of the Amide-I peak (1550–1720 cm^−1^) with the use of the Gaussian/Lorentzian profile three times in origin. The relative area of the best-fitted bands was used to calculate the relative proportion of each secondary structural component.

Sedimentation velocity (SV)^29^ experiments were carried out using a Beckman Optima L based UV-vis multiwavelength detector equipped Analytical Ultracentrifuge (AUC)^30–32^ with Ti double sector cells having 1.2 cm centerpieces (nanolytics GmbH, potsdam, D) and sapphire windows. Various concentrations of the protein and AuNC@Protein were dispersed in 100 mM NaCl at physiological pH and alkaline conditions for hydrodynamic investigations. To resolve the monomer-dimer formation, mass distributions, and non-ideality constants; the partial specific volumes (*μ̄*) were calculated by performing SV experiments in different salt solutions dissolved in 100% water and 100% heavy water (Table S1 and Fig. S1†) with density variation method.^33^ Buffer densities and viscosities at 20 °C were determined by using an oscillating U-tube (Anton Paar DMA 5000 M) and a HAAKE Falling Ball Viscometer C, respectively. All the SV experiments were performed at 20 °C and 50 krpm, with between 100–300 scans. The data were recorded for the protein at 280 nm and for AuNC@Protein at 320 nm for least-squares *g*(s), high-resolution sedimentation coefficient distribution *c*(s), and molar mass distribution *c*(M) with a resolution of 100–300 grid points and a confidence level of 0.96 by using SEDFIT algorithm.^34^

### Evaluation of peroxidase-like (POD-like) activity

The colorimetric assay parameters were optimized to obtain the maximum signal out of the AuNCs by changing the pH, the sodium chloride concentration, and the hydrogen peroxide concentration, and measuring the corresponding POD-like activity under these conditions. Protein-stabilized AuNCs were dialyzed against PBS overnight to remove excess NaOH and Au precursor. For the activity assay, 100 μL of H_2_O_2_ (5 M for AuNCs and 0.1 mM for HRP) and 100 μL of ABTS (1 mM for both AuNCs and HRP) were added to a 96-well plate. The enzyme, HRP, or AuNC (0.7 μM particle concentration, *ca.* 1.5 nm AuNC) solution was added and monitored at 415 nm in the time-scan mode for up to 30 min using a BioTek, Synergy plate reader at 25 °C. To investigate the kinetic mechanism of the enzymatic reaction, the concentration of H_2_O_2_ was varied and ABTS was kept constant and *vice versa*. The kinetic parameters were calculated based on the Michaelis–Menten equation^35^*ν* = *V*_max_ × [S]/(*K*_M_ + [S]), where *ν* is the initial velocity, *V*_max_ is the maximal reaction velocity, [S] is the concentration of substrate and *K*_M_ is the Michaelis constant.

## Results and discussion

### Characteristics of KTQ5C and AuNC@KTQ5C

**Fig. 2:**
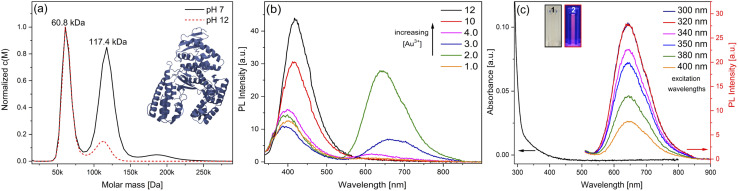
(a) Normalized *c*(*M*) *versus* molar mass distribution of KTQ5C (*μ̄* = 0.728 mL g^−1^) at pH 7 and 12, the inset presents the predicted 3D conformation of KTQ5C, (b) fluorescence spectroscopy of AuNC@KTQ5C with a constant protein and an increasing gold concentration under an excitation wavelength of 320 nm, (c) UV-visible and fluorescence spectroscopic analysis of AuNC@KTQ5C (4 mg mL^−1^ KTQ5C: 2 mM HAuCl_4_), the insets include photographs of the AuNC@KTQ5C in (1) visible light and (2) 365 nm UV light. The UV/vis signal with broad absorption is on the left side and the maximum emission peak of the PL intensity centering at 640 nm for all investigated excitation wavelengths is on the right.

In this work, the thermostable protein KlenTaq was designed as AuNC stabilizing agent by introducing five cysteine residues (KTQ5C) located in the protein nanopocket. The purity of the recombinant protein was demonstrated by Matrix-Assisted Laser Desorption/Ionization (MALDI-TOF) and SDS-PAGE analysis as well as the thermostability up to 83 °C as probed by dynamic light scattering size measurements (Fig. S2†). The hydrodynamic resolution of KTQ5C in SV experiments can provide additional information compared to SDS-PAGE and MALDI-TOF because the analysis reveals the distribution of the monomer, dimer, and trimer of KTQ5C in buffer conditions. Therefore, to investigate the homogeneity of the protein, we performed SV experiments in the analytical ultracentrifuge (AUC). The *c*(*M*) distribution displayed in Fig. 2a, not only shows that KTQ5C mostly forms monomers (65.2%) at pH 12 and mostly dimers (55%) at pH 7 but also claims that the monomeric form of KTQ5C interacts with metal precursor during the synthesis of AuNCs, which is carried out at pH 12. To gain structural insights into KTQ5C, the AlphaFold^36^ algorithm was utilized to predict the 3D conformation, shown as an inset in Fig. 2a. The result indicates that KTQ5C and KlenTaq (Fig. 1) adopt almost the same structure with slight differences in the cleft and protected protein nanopocket for efficient stabilization of AuNCs by providing a metal-coordination and accumulation scaffold.

The nucleation and growth mechanism of AuNCs in a protein *via* the bio-inspired synthesis route is highly dependent on the structural and compositional features of the protein.^37,38^ Besides, it has been proven that the photoemission mechanism of protein-stabilized AuNCs is affected by initial gold concentration.^39^ Therefore, to display the efficient conditions for the protection and stable emission of AuNCs in designed KTQ5C, optimization reactions (Fig. 2b) with measurement of the HAuCl_4_ concentration-dependent fluorescence of AuNC@KTQ5C were performed. The results showed that increasing the amount of gold precursor from 4 mM to 12 mM brought out a red shift in intrinsic emission maxima of the aromatic side chains from 390 nm to 416 nm which could be due to protein partial unfolding^40,41^ and an increased density of polar protein microenvironment around tryptophan (Trp) or interaction of hydrophilic side chains with Trp.^42^ The decrease in the autofluorescence of the aromatic side chains in combination with the increment in the emission maxima at 640 nm indicates prominent stabilization of red-emitting AuNCs in KTQ5C. The emission peak located at 390 nm corresponds to the presence of Au_8_NC@KTQ5C^38,43,44^ which can be the reaction intermediate in the nucleation and growth process of the fluorescent AuNCs. Hence, to stabilize the fluorescent AuNCs in the presence of KTQ5C, the reaction condition with a HAuCl_4_ concentration of 2 mM was applied for further experiments.

In contrast to larger nanoparticles, AuNCs do not exhibit surface plasmon resonance peaks but rather have molecular-like absorption and fluorescence properties owing to discrete energy levels and ultra-small sizes.^45,46^Fig. 2c depicts the absorption and fluorescence spectra of AuNC@KTQ5C, with the maximum emission peak centers at 640 nm upon various excitation wavelengths. The unchanged position of the emission peak upon different excitations indicates the stability and real emission response of the AuNC@KTQ5C. The maximum emission intensity with the excitation wavelength of 320 nm claims the maximum absorption band of the red-emitting AuNC@KTQ5C. Additionally, the emission peak around 350–450 nm as shown in Fig. 2b shifts to a longer wavelength and decreases in intensity under lower energy excitations (Fig. S3†) which demonstrates that those peaks do not correspond to the fluorescent entities but to the Raman signal of the reaction intermediates.

Moreover, the high-resolution transmission electron microscope (HR-TEM) image and size distribution analysis of AuNC@KTQ5C (Fig. S4a and b†) depict that the average size of the AuNCs is 2.6 ± 0.3 nm. MALDI-TOF mass spectra of KTQ5C and AuNC@KTQ5C (Fig. S4c†) reveal the encapsulation of the reaction intermediate Au_8_ and stable Au_20_ in KTQ5C as well. Au_20_ tetrahedral nanocluster is literature-known and described to be very stable.^47^ However, the molar mass distribution of AuNC@KTQ5C as a result of AUC experiments is continuous and broad with molar masses up to 400 kDa (Fig. S4d†) due to the size distribution of the AuNCs (Fig. S4b and c†), which causes a molar mass distribution as well as a density distribution of the AuNC@KTQ5C. Therefore, the protein hydrodynamics before and after the stabilization of AuNCs were analyzed by plotting 1/*s*_w_ (inverse the weighted-average sedimentation coefficient) *versus* concentration. This generally gives a more useful extrapolation than that of *s*_w_*vs. c* (Fig. S5†). Table 1 shows *s*_w_ at infinite dilution (*s*^0^_w_) and hydrodynamic non-ideality coefficient (*k*_s_) values for KTQ5C and AuNC@KTQ5C under pH 7 and 12. Sedimentation of KTQ5C under different pH conditions shows a slightly decreased value of *s*^0^_w_ at an alkaline environment indicating almost no effective mass changes of KTQ5C, which would result in the slow or fast sedimentation of the protein in the solution. On the other hand, the difference in the *k*_s_ is caused by a combination of excluded volume and shape effects,^48,49^ indicating varying degrees of hydration and slight conformational changes of KTQ5C under different conditions. On top of that, stabilization of AuNC in KTQ5C resulted in higher *s*^0^_w_ and *k*_s_ values pointing to the efficient synthesis of AuNCs in the protein and also conformational changes and hydration to a degree that is more prominent at alkaline conditions.

**Table 1:** Hydrodynamic parameters of KTQ5C and AuNC@KTQ5C^a^

	pH	*s* ^0^_w_ [S]	*k*_s_ [L g^−1^]
KTQ5C	7	4.15	0.0009
	12	4.11	0.0040
AuNC@KTQ5C	7	6.71	0.0554
	12	5.26	0.1304

a *s* ^0^_w_: weighted-average sedimentation coefficient at infinite dilution, *k*_s_: hydrodynamic non-ideality constant, (*k*_s_/1/*s*^0^_w_ = slope of the 1/*s*_w_*vs.* conc. plot).

### Thermostability of AuNC@KTQ5C

**Fig. 3:**
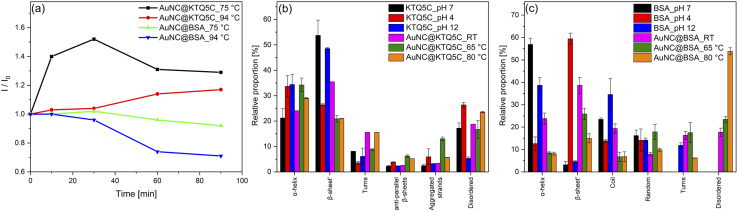
(a) Thermal fluorescence stability assay of AuNCs at 75 °C and 94 °C for 90 min and deconvolution histograms of the Amide-I peak corresponding to (b) KTQ5C and (c) BSA at the physiological state of the environment, at pH 4, pH 12, and protein–AuNCs conjugates before and after the heat treatment.

Having established that the thermostable KTQ5C can stabilize fluorescent AuNCs, we sought to investigate whether the AuNC@KTQ5C was also thermostable in terms of fluorescent emission efficiency. To compare the stability of the AuNC@KTQ5C with the widely utilized fluorescent counterpart, AuNCs were synthesized in bovine serum albumin (BSA) (AuNC@BSA) and characterized (Fig. S6 and S7†). Heat treatment results (Fig. 3a) of AuNCs stabilized with KTQ5C and BSA indicated that AuNC@KTQ5C had no decrease in fluorescent emission at 75 °C and 94 °C which confirms the stable protein-AuNCs interaction at high temperatures. Besides, the enhanced fluorescence intensity of AuNC@KTQ5C at high temperatures would be due to decrement in AuNCs–AuNCs distances owing to the slight conformational changes of the protein.

Oppositely, AuNC@BSA lost 30% of the emission intensity after heat treatments. This could be caused by the denaturation of BSA after heat treatment, which results in the deformation of the interactions between protein and AuNCs. To further investigate the effect of AuNCs formation and the heat treatments on the protein secondary structure, the Amide-I band in ATR-FTIR spectra was curve-fitted and deconvoluted into α-helices, β sheets, turns, coils, aggregated strands, and disordered structures.^50,51^Fig. 3b demonstrates that KTQ5C has resistance to change in the percentage of α-helices and does not have increased percentages of disordered structures either in AuNCs synthesis or after the heat treatments. Yet, as shown in Fig. 3c, for BSA a high amount of disordered structures is formed after reduction reactions and heat treatments.

Moreover, we also found high biocompatibility of the AuNC@KTQ5C by proving that they were non-toxic to HeLa cells up to 200 μg mL^−1^ concentration of AuNCs (Fig. S8†) making them employable in biological applications.

Taken together, these results demonstrate that KTQ5C enables not only the biocompatible and functional stabilization of fluorescent AuNCs but also, up to our knowledge, the first-time thermostable protein-stabilized nanoclusters.

### Assessment of the enzymatic activity

Because of the well-known catalytic activity of ultra-small nanoclusters,^21,52^ the thermostability of the as-synthesized AuNC@KTQ5C caught our attention to assess its efficacy as an enzyme mimic at high temperatures where the natural counterparts have limitations. Therefore, oxidation of the POD substrate ABTS by H_2_O_2_ was employed as a model catalytic reaction to investigate the POD-like activity of the AuNC@KTQ5C. The absorbance readout at 415 nm (A_415_) provided a colorimetric result for the calculations of steady-state kinetic parameters of AuNC@KTQ5C and the counterparts. In deciding, which reaction conditions are optimal to reach a maximum signal, we conducted optimization experiments and chose a buffer solution having a pH of 4 and sodium chloride concentration of 0.4 M at room temperature (Fig. S9†). To achieve a better comparison between the natural enzyme horseradish POD (HRP), AuNC@BSA, and thermostable AuNC@KTQ5C in terms of their enzymatic efficacies, they were all examined under the same reaction conditions described above. However, HRP had to be used at a much lower concentration than AuNC@BSA and AuNC@KTQ5C leading to its high *K*_cat_ in Table 2.

**Table 2:** Comparison of the kinetic parameters of AuNC@KTQ5C, AuNC@BSA, and HRP in the presence of ABTS and H_2_O_2_ as substrates at pH 4 and RT^a^

	*c*_enzyme_ [M]	Substrate	*K*_M_ [mM]	*V*_max_ [M s^−1^]	*K*_cat_ [s^−1^]
AuNC@KTQ5C	6.6 × 10^−8^	ABTS	0.062	34.0 × 10^−9^	0.52
AuNC@KTQ5C	6.6 × 10^−8^	H_2_O_2_	0.040	9.30 × 10^−9^	0.14
AuNC@BSA	9.2 × 10^−8^	ABTS	0.206	7.18 × 10^−9^	0.08
AuNC@BSA	9.2 × 10^−8^	H_2_O_2_	0.070	2.61 × 10^−9^	0.03
HRP	3.5 × 10^−11^	ABTS	0.383	2.57 × 10^−9^	0.73 × 10^2^
HRP	3.5 × 10^−11^	H_2_O_2_	0.035	0.67 × 10^−9^	0.19 × 10^2^

a *c*_enzyme_: total enzyme (or AuNC) concentration, *K*_M_: Michaelis constant, *V*_max_: maximal reaction velocity, *K*_cat_: catalytic constant/turnover number that equals *V*_max_/*c*_enzyme_.

**Fig. 4:**
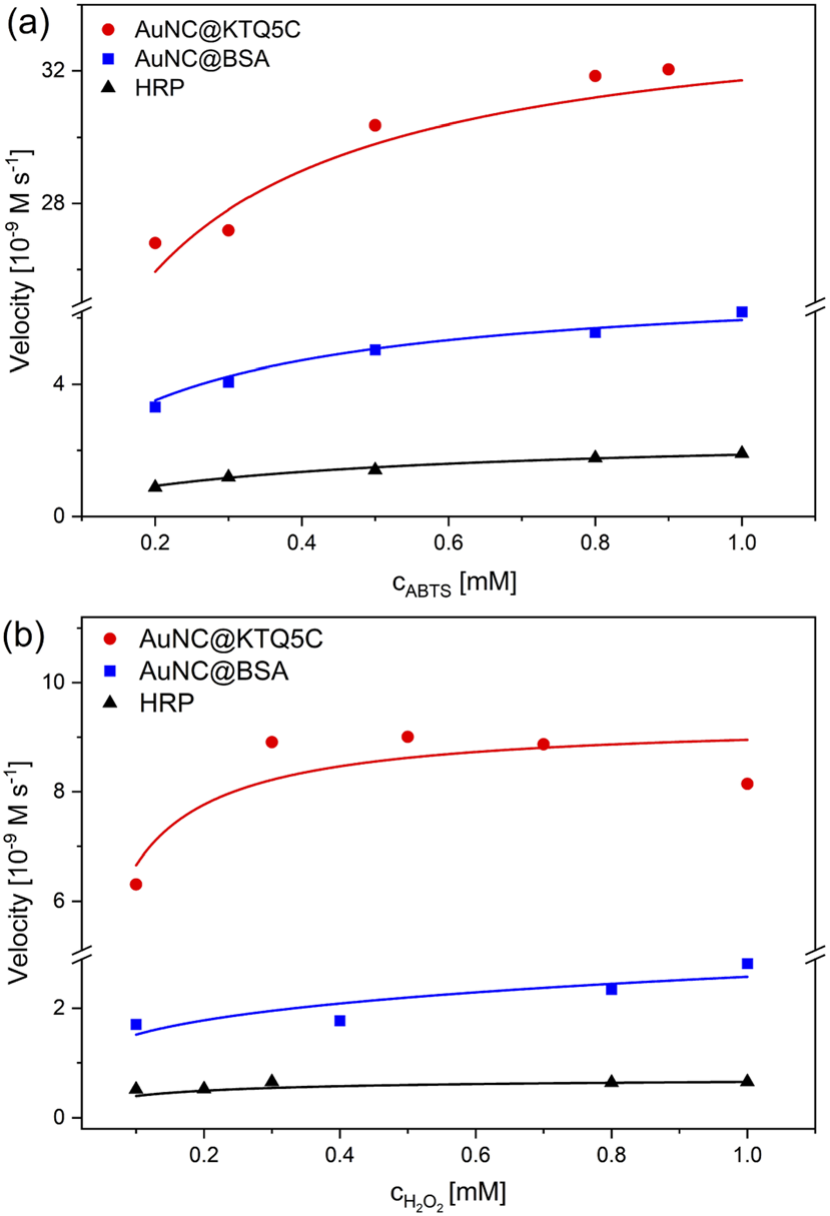
Steady-state kinetic assays of three counterparts; AuNC@KTQ5C, AuNC@BSA, and HRP. Plots of velocity against (a) ABTS concentration, in which H_2_O_2_ was fixed and (b) H_2_O_2_ concentration with a constant ABTS concentration.

The absorbance data obtained at certain time intervals with varying concentrations of ABTS and H_2_O_2_ for all counterparts were fitted to the Michaelis–Menten model (Fig. 4). The fitted curves for all three enzymes exhibit typical Michaelis–Menten kinetics. The steady-state kinetic parameters given in Table 2 show that the Michaelis constant (*K*_M_) value of AuNC@KTQ5C (0.062 mM) with ABTS as the substrate is about six times lower than that for HRP and three times lower than that for AuNC@BSA, suggesting that AuNC@KTQ5C may have a higher affinity for ABTS than HRP and AuNC@BSA.

The apparent high *V*_max_ values for AuNC@KTQ5C as 34.0 × 10^−9^ M s^−1^ and 9.30 × 10^−9^ M s^−1^ indicate more efficient enzyme–substrate saturation in comparison to AuNC@BSA and HRP, which is also a consequence of the high enzyme–substrate tendency. In addition, no inhibition was found for the AuNCs-catalyzed reaction at a H_2_O_2_ concentration in the mM range (0.1–1.0 mM), suggesting that AuNCs exhibit stable catalytic activity at high H_2_O_2_ concentration in comparison to the natural enzyme HRP. The HRP activity at a lower H_2_O_2_ concentration is consistent with the lower *K*_M_ value of HRP (0.007 mM) towards the substrate (Fig. S10 and Table S2†).

The efficient POD-like activity of AuNC@KTQ5C is likely due to the positively charged protein shell (Fig. S11†) interacting electrostatically with the negatively charged substrate at pH 4. Additionally, the Au_20_NCs core in KTQ5C is known as a magic-sized cluster having a large energy gap (1.77 eV) and interesting catalytic activities due to its unique and high symmetry structures.^53–55^ On top of that, the enzymatic efficacy difference between AuNC@KTQ5C and AuNC@BSA also indicates that the size regime of AuNCs stabilized in the protein affects the overall activity of the nanozyme, which is more efficient in the case of AuNC@KTQ5C having a smaller number of Au (20) in comparison to AuNC@BSA (25) owing to surface-volume ratio^56^ getting larger for smaller sizes. It is also worth noting that, in comparison with other reported nanoparticles and nanoclusters exhibiting POD-like activities towards the same substrates (Table S3†), AuNC@KTQ5C had the smallest *K*_M_ value towards ABTS, which may be due to the higher or more retentive AuNC surface–substrate interaction provided by the stabilization of AuNCs in the protein dimer (Fig. S3d†), nanopocket, which may have a cage or nanoreactor effect. Since the *V*_max_ value of an enzyme is highly dependent on the environmental conditions (temperature, pH, and ionic strength), the systems reported in Table S3† are not comparable with AuNC@KTQ5C in terms of the *V*_max_. In addition, POD-mimicking AuNC@KTQ5C comes into prominence from nanoparticle-based enzyme mimics not only with having a higher tendency to the substrate but also requiring a single-step environmentally benign synthesis. Moreover, the nanoparticle-based enzyme candidates are notoriously prone to aggregation, particularly in *in vivo* applications, while AuNC@KTQ5C has attracted attention through their appropriate size regime promising non-toxicity by renal clearance.

### Comparison of robustness and reusability of AuNC@KTQ5C, AuNC@BSA, and HRP

**Fig. 5:**
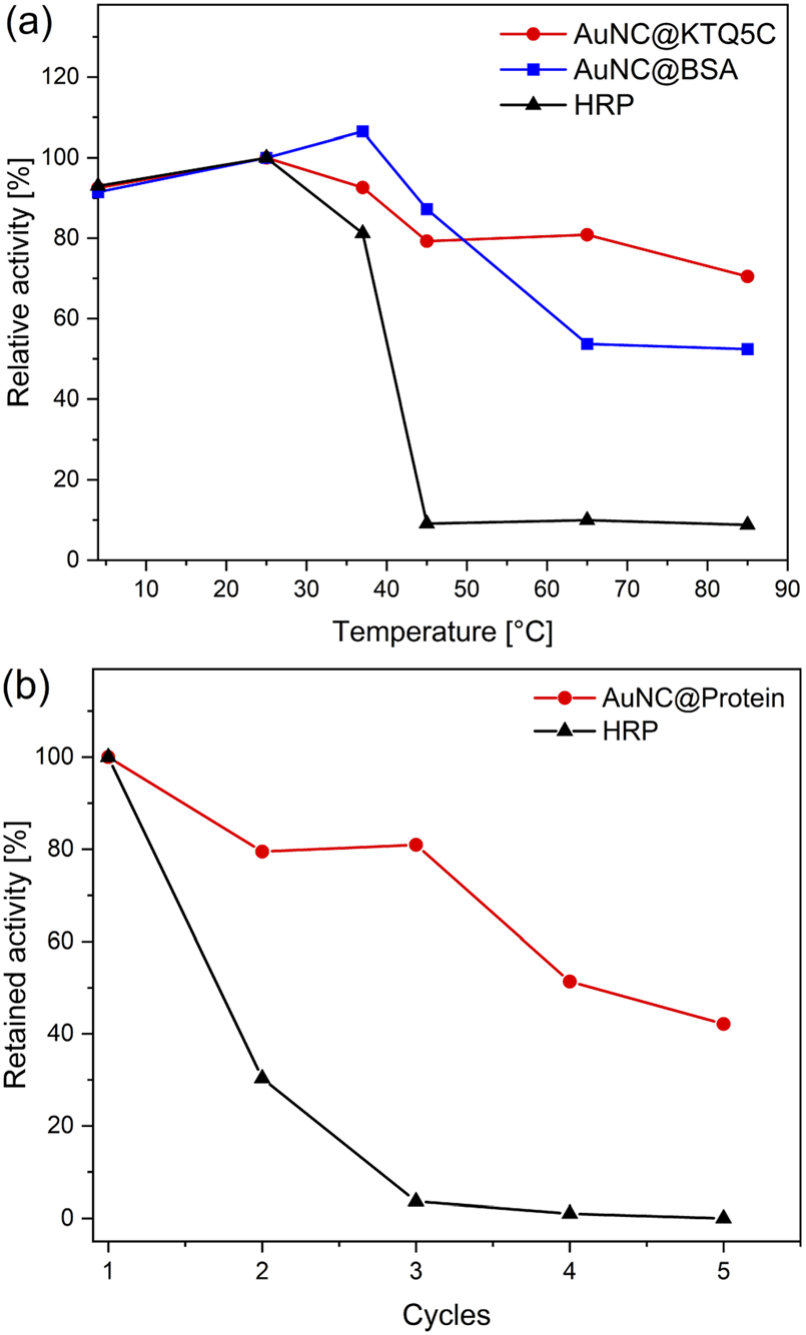
(a) Measurement of the relative enzymatic activity of AuNC@KTQ5C, AuNC@BSA, and HRP after incubation at a range of temperatures from 4 to 85 °C for 2 h. (b) Relative enzymatic activity of protein-stabilized AuNCs and HRP after five cycles.

To ensure the robustness of their enzymatic activities, AuNC@KTQ5C, AuNC@BSA, and HRP were incubated for 2 h at a range of temperatures (4–85 °C), and relative activities were examined under optimized conditions (at pH 4 and 25 °C). As displayed in Fig. 5a, the POD-like activity of AuNC@KTQ5C was retained (above 70–80% activity) over a wide range of temperatures, while AuNC@BSA lost half of its activity after incubation at a denaturation temperature of 60 °C. On the other hand, treatments at temperatures above 40 °C resulted in a complete loss of enzymatic activity of HRP. Thus, the higher POD-mimicking activity of AuNC@KTQ5C under harsh reaction temperatures is demonstrated. Additionally, the reusability of HRP and AuNC@KTQ5C were also tested by separating the enzyme from the reaction mixture by ultra-filtration (membrane size of 10 kDa cut off) and using it again in the next catalysis reaction. As demonstrated in Fig. 5b, AuNC@KTQ5C exhibits a considerably better performance than HRP after 5 cycles of enzymatic reaction.

## Conclusions

In this study, we have provided the first report in which a thermostable protein has been designed to stabilize biocompatible, fluorescent, and ultra-small AuNCs. The structural features and integrity of KTQ5C have been found to favor the formation of magic-sized Au_20_NCs. Owing to the rigidity of KTQ5C, AuNC@KTQ5C has exhibited thermostable photoluminescence with a conserved amount of secondary structural components after heat treatment. Besides the preserved photoluminescence at high temperatures, it has also been proven that AuNC@KTQ5C possesses efficient POD-like enzymatic activity following typical Michaelis–Menten kinetics, demonstrating higher affinity and reusability in the presence of ABTS as the substrate compared to that of natural POD. Moreover, the thermostable behavior of KTQ5C has also ensured the catalytic performance of AuNC@KTQ5C at high-temperature reaction conditions with sufficient activity.

Exhibiting heat-resistant enzymatic activity benefits AuNC@KTQ5C to carry out enzymatic reactions at higher temperatures, thus, boosting conversion rates and substrate solubility and reducing the risk of microbial growth and the viscosity of the reaction medium. Besides, since protein–encapsulated AuNCs have been implemented in bio-friendly light-emitting materials^57,58^ and theranostic applications^59,60^ in which local temperatures would arise, we envision that our designed strategy having enhanced thermal stability would lead to new approaches and formulations of AuNCs in these fields. Besides, stabilization of the Au_20_NC core in the protein cage can be applicable in other catalytic reactions that demand high-performance, stable, and biocompatible nanozymes.

## Author contributions

The manuscript was written through the contributions of all authors./All authors have given approval to the final version of the manuscript.

## Conflicts of interest

There are no conflicts to declare.

## Supplementary Material

NA-005-D3NA00566F-s001
