# Supplementary material for: Thermostable protein-stabilized gold nanoclusters as a peroxidase mimic
Source: Nanoscale Adv. 2023 Sep 21;5(22):6061–8. doi: 10.1039/d3na00566f (PMC10629000; doi:10.1039/d3na00566f)
Supplement: NA-005-D3NA00566F-s001 [file NA-005-D3NA00566F-s001.pdf]

Electronic Supplementary Material (ESI) for Nanoscale Advances.
This journal is © The Royal Society of Chemistry 2023

Supporting Information

Thermostable Protein-Stabilized Gold Nanoclusters as a Peroxidase Mimic

Özlem Akyüz, Maite Mißun, Rose Rosenberg, Martin Scheffner, Andreas Marx, Helmut Cölfen*

Density Variation Method in AUC[1]

The average partial specific volumes ($\bar{v}$) of the protein and protein-nanocluster conjugates were evaluated by using the density variation sedimentation analysis by taking into account an average molar mass ratio of proteins in 100 % deuterated and in non-deuterated solvents ($k = 1.0155$)[2,3] due to the H-D exchange of hydrogens in the protein by deuterium.

Here, R represents the effect of density variation on the sedimentation of biomolecules with η as the solvent viscosity, s as the sedimentation coefficient, and the D and H as the subscripts indicating H_2O and D_2O solvent, respectively.

$$R = \frac{s_D \eta_D}{s_H \eta_H}$$

$$\bar{v} = \frac{k - R}{\rho_D - R\rho_H}$$

Table S1: Density and viscosity of the solutions used in sedimentation velocity experiments at 20 °C.

	**Density (ρ) [g/mL]**		**Viscosity (η) [mPa s]**	
Solution	Aqueous	Heavy water	Aqueous	Heavy water
0.2 M NaCl	1.006	1.110	0.946	1.101
0.5 M NaCl	1.018	1.128	1.038	1.277
1 M NaBr	1.076	1.181	1.055	1.519

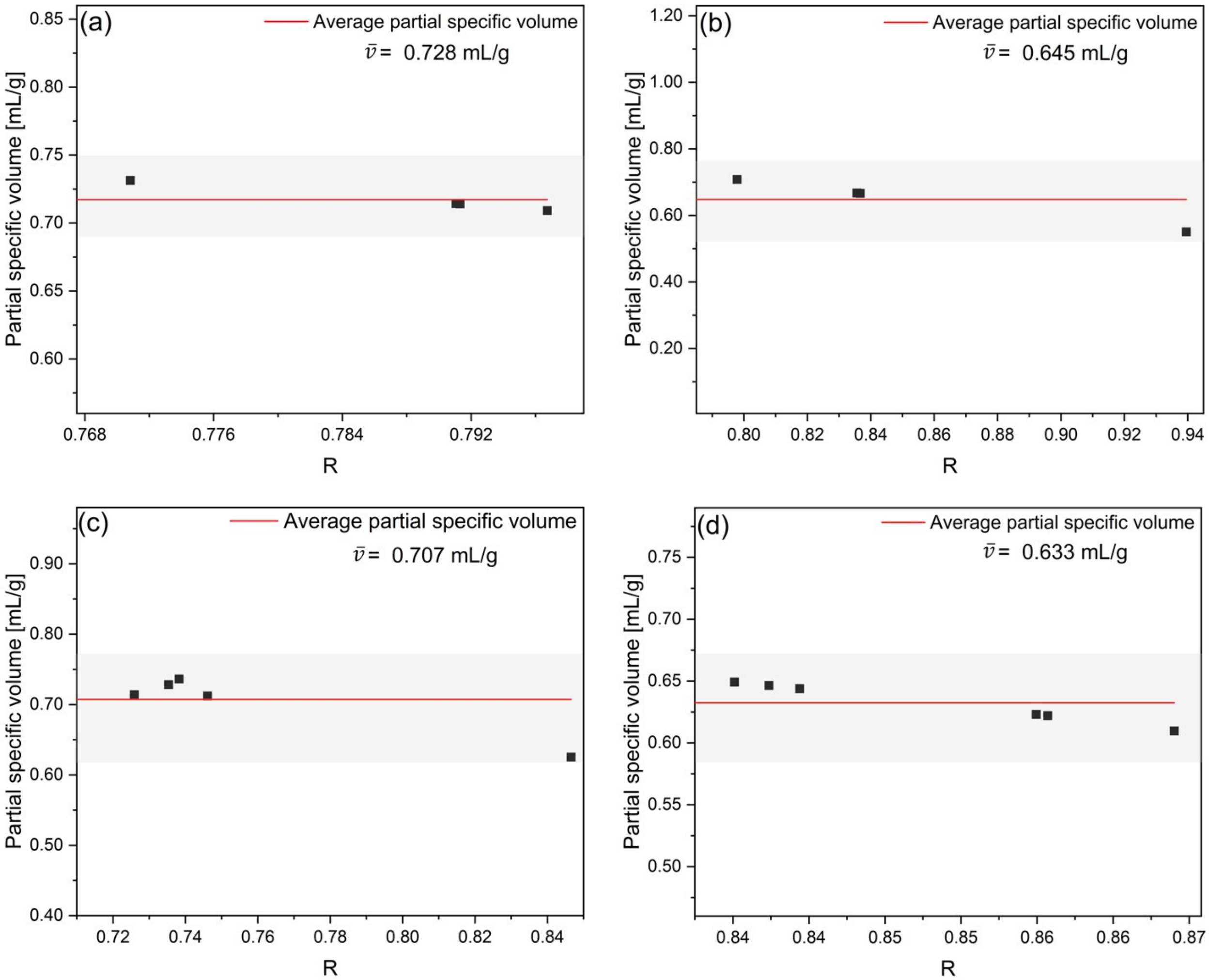

Fig. S1. Partial specific volume calculations for a) KTQ5C, b) AuNC@KTQ5C, c) BSA, and d) AuNC@BSA with density variation method.

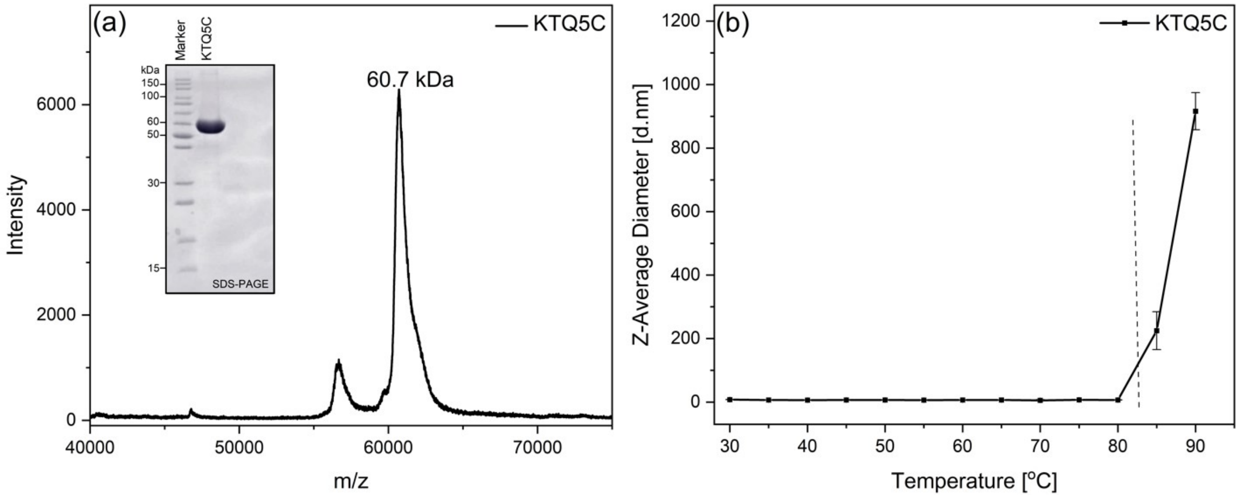

Fig. S2. a) Matrix-assisted laser desorption/ionization–time-of-flight (MALDI-TOF) mass spectrometry analysis and Coomassie-stained SDS-PAGE image of the cysteine-modified KlenTaq (KTQ5C) confirms the purification of the desired protein. b) Thermal ramping analysis of KTQ5C in Tris-HCl buffer at pH 8.55 by Dynamic Light Scattering (DLS) shows the thermal stability of KTQ5C up to 83 °C.

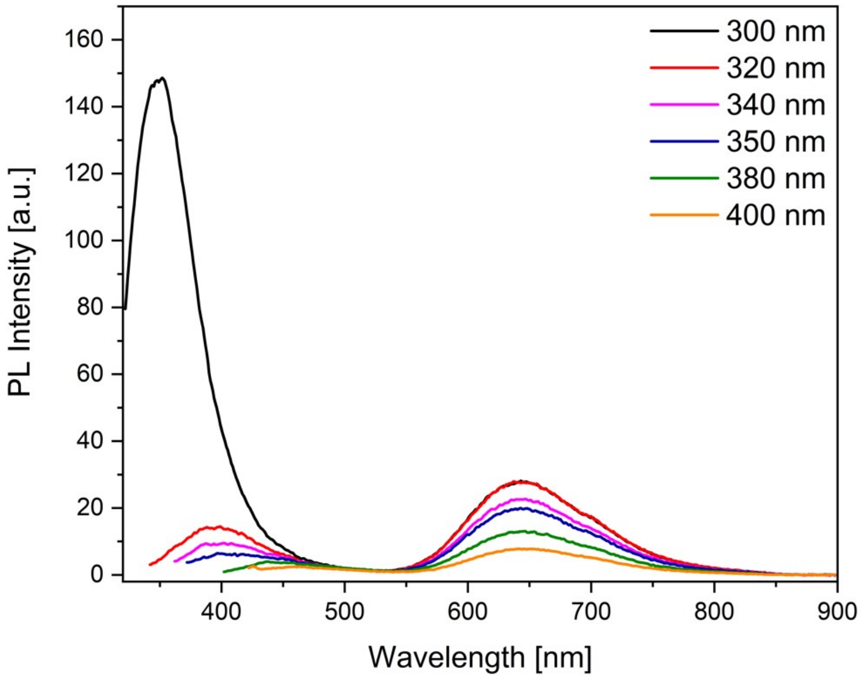

Fig. S3. Fluorescence spectroscopy of KTQ5C stabilized gold nanoclusters (AuNC@KTQ5C) under different excitation wavelengths (300- 400 nm). The peak located at 350 nm shifts to the longer wavelength and is broadened. On the other hand, the emission maxima at 640 nm remained unchanged upon different excitation wavelengths.

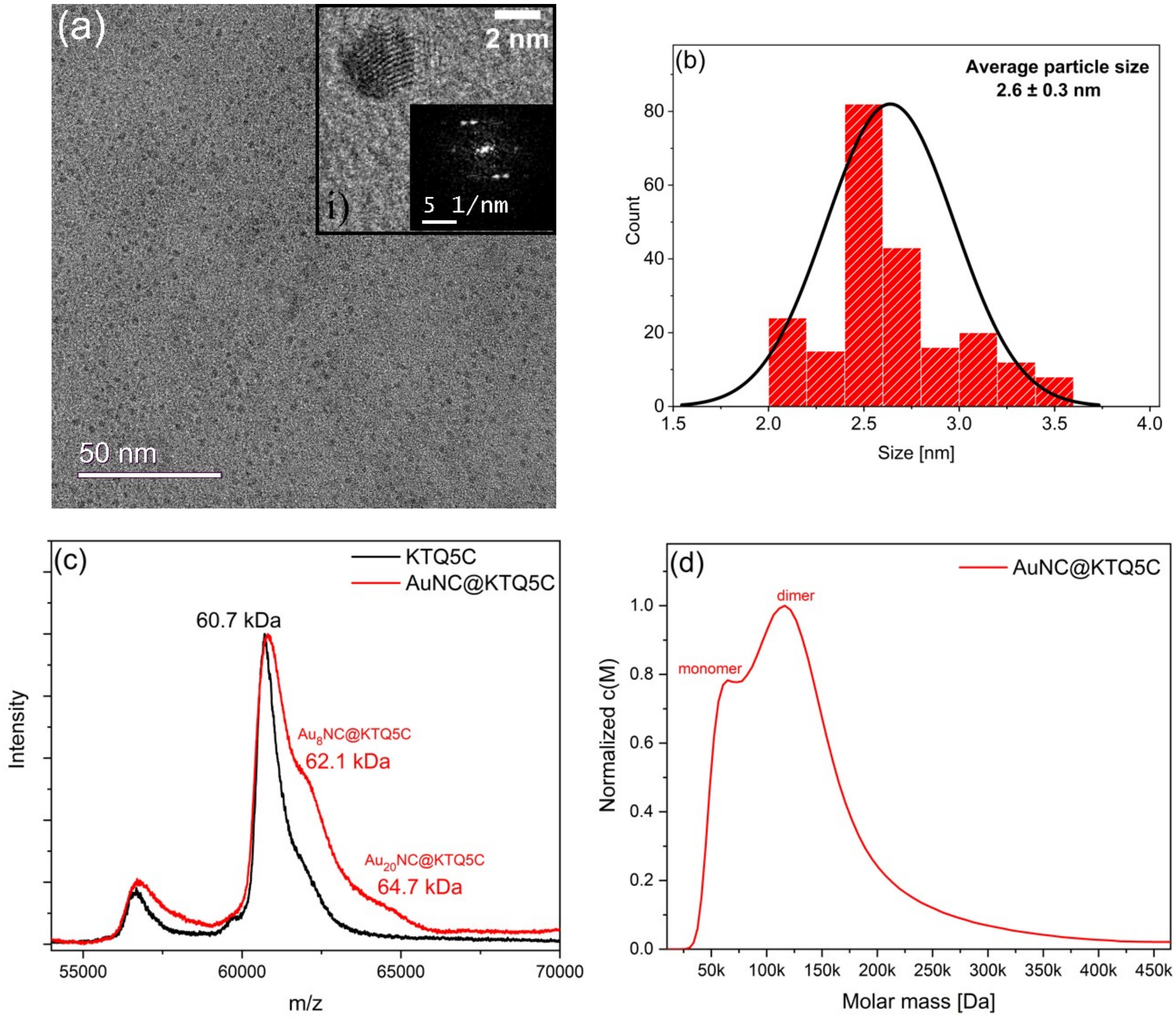

Fig. S4. a) HR-TEM images of AuNC@KTQ5C at low and i) high-magnification with corresponding Fast Fourier Transform (FFT) image revealing the presence of lattice fringes of AuNCs, b) Size distribution of AuNC@KTQ5C is presented in a histogram. The average size and error are derived from the maximum respectively the half-width of the Gauss fit of the data, c) MALDI-TOF mass spectrometry analysis of AuNC@KTQ5C demonstrates the presence of reaction intermediate Au_8NC and red-emitting magic-sized $Au_{20}NC$, d) Normalized c(M) vs mass distribution of AuNC@KTQ5C ($\bar{v}$ = 0.645 mL/g) at pH 7 from Analytical Ultracentrifugation (AUC) sedimentation velocity analysis indicating the formation of mostly dimeric KTQ5C encapsulated AuNCs and the broad mass distribution (40 kDa- 400 kDa) of AuNC@KTQ5C.

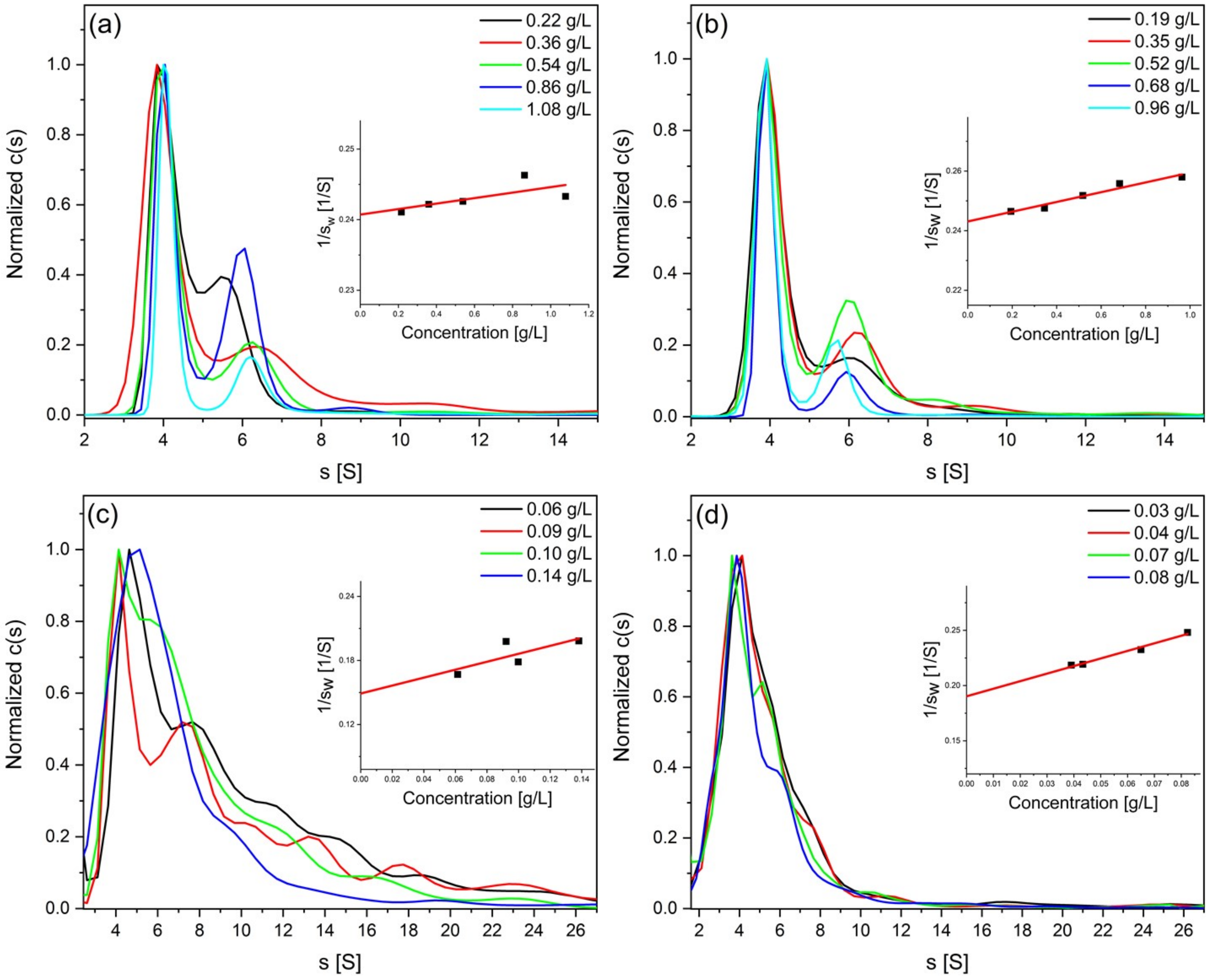

Fig. S5. Hydrodynamic analysis of KTQ5C and AuNC@KTQ5C (dissolved in 100 mM NaCl (ρ = 1.00176 g/mL and η = 0.907 mPa s)). Diffusion corrected sedimentation velocity, *c(s)* analysis of KTQ5C at a) pH 7 (conc. range 0.22 - 1.08 g/L) and b) pH 12 (conc. range 0.19 - 0.96 g/L), and AuNC@KTQ5C at c) pH 7 (conc. range 0.06 - 0.14 g/L) and d) pH 12 (conc. range 0.03 - 0.08 g/L). A linear fit was performed to calculate the weighted-average sedimentation coefficient at infinite dilution (s^0_w) by extrapolation of $1/s_w$ to zero concentration (inset). Slope of the curve ($1/s_w$ vs conc.) was used to calculate the non-ideality constant (k_s).

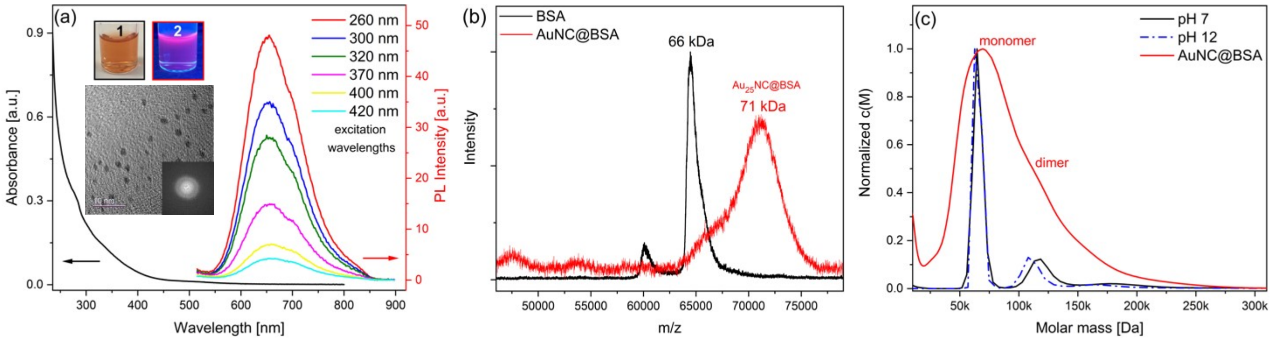

Fig. S6. a) UV-visible and fluorescence spectroscopic analysis of AuNC@BSA synthesized *via* the method reported by Xie et al.[4] Briefly, 20 mg/mL of BSA solution was mixed with 10 mM $HAuCl_4$, after 5 minutes of incubation, NaOH was added to reach the pH of 12 and subsequently incubated at 37 °C overnight. PL spectra indicate that AuNC@BSA possess emission wavelength of 650 nm upon different irradiation wavelengths, the insets include photographs of the AuNC@BSA in (1) visible light and (2) 365 nm UV light. The inset HR-TEM image belongs to AuNC@BSA and displays ultra-small sized entities around 2 nm. b) MALDI-TOF mass spectrometry analysis of AuNC@BSA demonstrates the stabilization of red-emitting Au_{25}NC, the peak at around 60 kDa for BSA is due to presence of some impurity in the protein sample itself, c) Normalized c(M) mass distribution of BSA ($\bar{v} = 0.707\ mL/g$) at pH 7 and 12 and AuNC@BSA ($\bar{v} = 0.633\ mL/g$) at pH 7 from AUC sedimentation velocity analysis indicating the formation of mostly monomeric BSA encapsulated AuNCs and the broad mass distribution (20 kDa- 250 kDa) of AuNC@BSA.

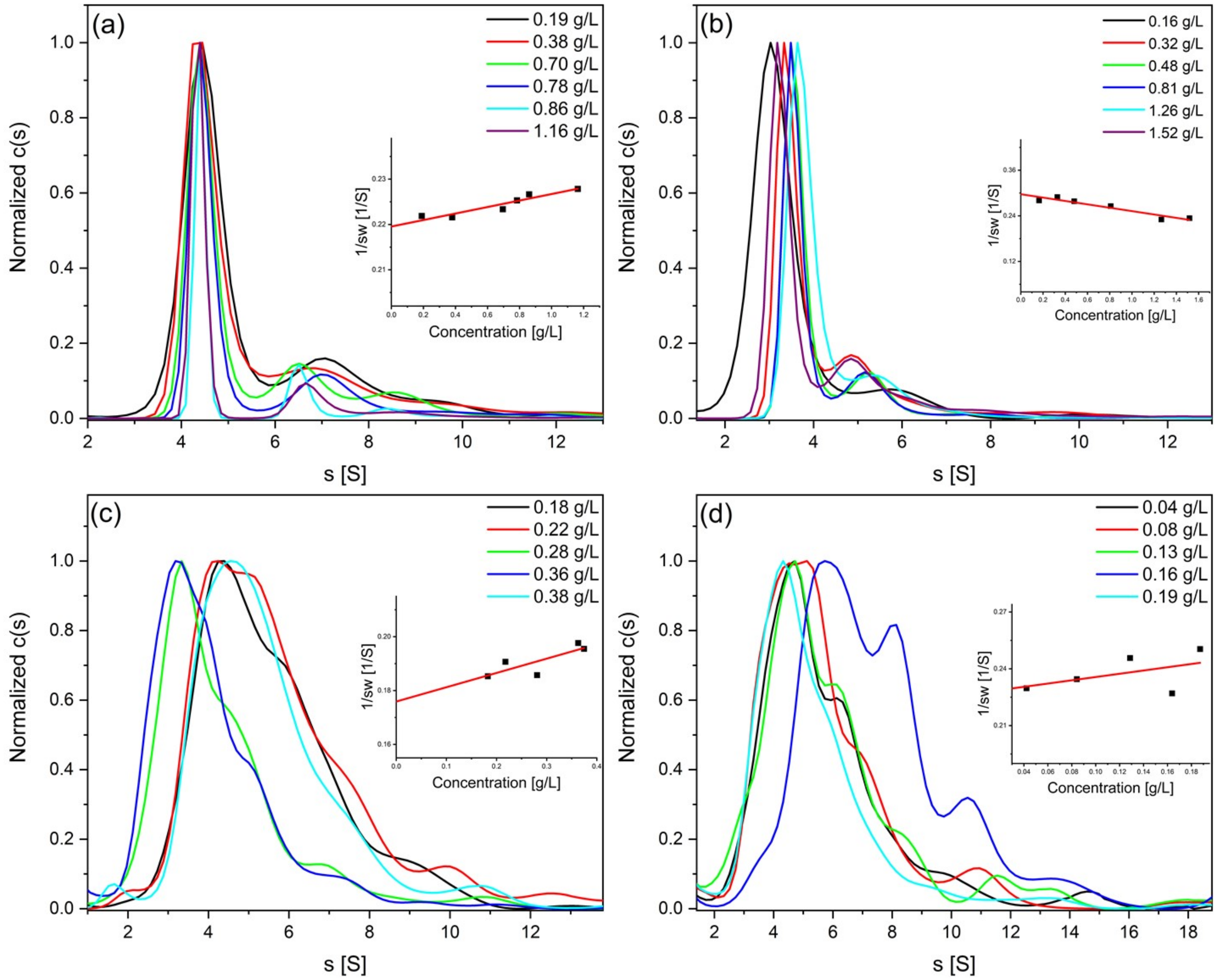

Fig. S7. Hydrodynamic analysis of BSA and AuNC@BSA (dissolved in 100 mM NaCl). Sedimentation velocity, *c(s)* analysis of a) BSA at pH 7 (conc. range 0.19 - 1.16 g/L) with the graph of the inverse weighted-average sedimentation coefficient, $1/s_w$ as a function of BSA concentration (inset). Y-intercept gives the weighted-average standardized sedimentation coefficient at infinite dilution (s^0_w) value of 4.55 S and slope gives the thermodynamic non-ideality constant, k_s (k_s/ $1/s^0_w$= slope) which is 0.0016 L/g. b) BSA at pH 12 (conc. range 0.16 - 1.52 g/L) with the s^0_w value of 3.37 S and k_s of -0.0132 L/g. c) AuNC@BSA at pH 7 (conc. range 0.18 - 0.38 g/L) with the s^0_w value of 5.68 S and k_s of 0.0093 L/g. d) AuNC@BSA at pH 12 (conc. range 0.04 - 0.19 g/L) with s^0_w value of 4.40 S and k_s of 0.0196 L/g.

Cell Viability Assay

In our cell viability experiments, a thiazolyl blue tetrazolium bromide (MTT) assay was used. Human cervical cancer (HeLa) cells dispersed in Dulbecco's modified eagle medium (DMEM), supplemented with 10 % fetal bovine serum, 100 U of penicillin, and 100 µg/mL streptomycin were seeded in 96-well plates at a density of 1×10^4 cells per well. After 24 h incubation at 37 °C in 5 % CO_2, the media were refreshed and AuNC@KTQ5C and AuNC@BSA in different concentrations were added into the wells and further incubated overnight. Three replicate samples were prepared for each concentration in three different well plates. The MTT solution (12 mM, 20 µL in PBS) was added to each well and incubated for 4 h at 37 °C and 5 % CO_2. Then, the medium was removed and 250 µL dimethyl sulfoxide (DMSO) was added to dissolve formazan crystals. After shaking of the plates for 30 min at room temperature, the absorbance at 570 nm was measured using a microplate reader. Viability was determined as a percentage of control (viability of control cells was set as 100 %). Absorbance values were corrected with blank.

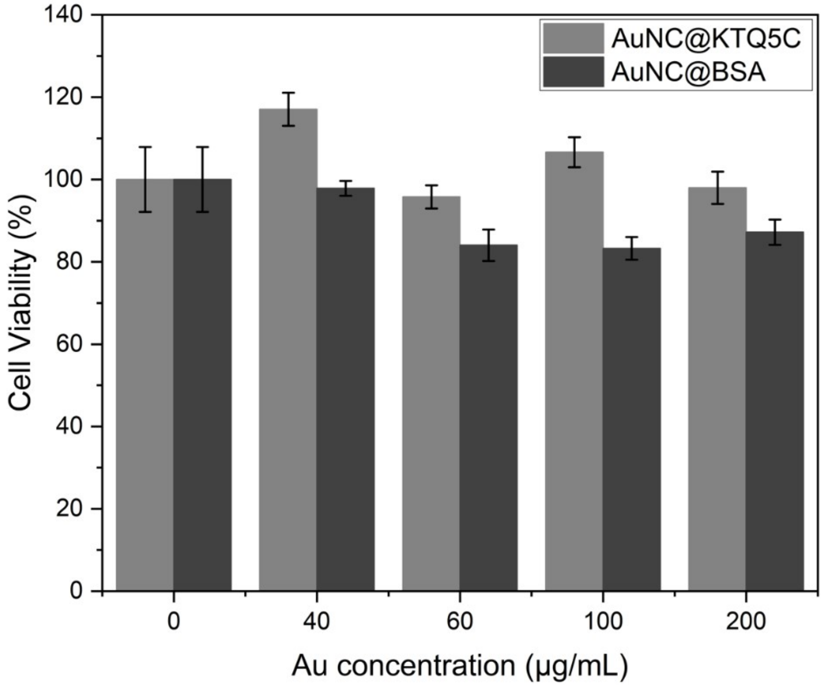

Fig. S8. Viability of HeLa cells after 24 h of incubation with different concentrations of KTQ5C and BSA stabilized AuNCs. The error bars represent the fluctuations among three independent measurements.

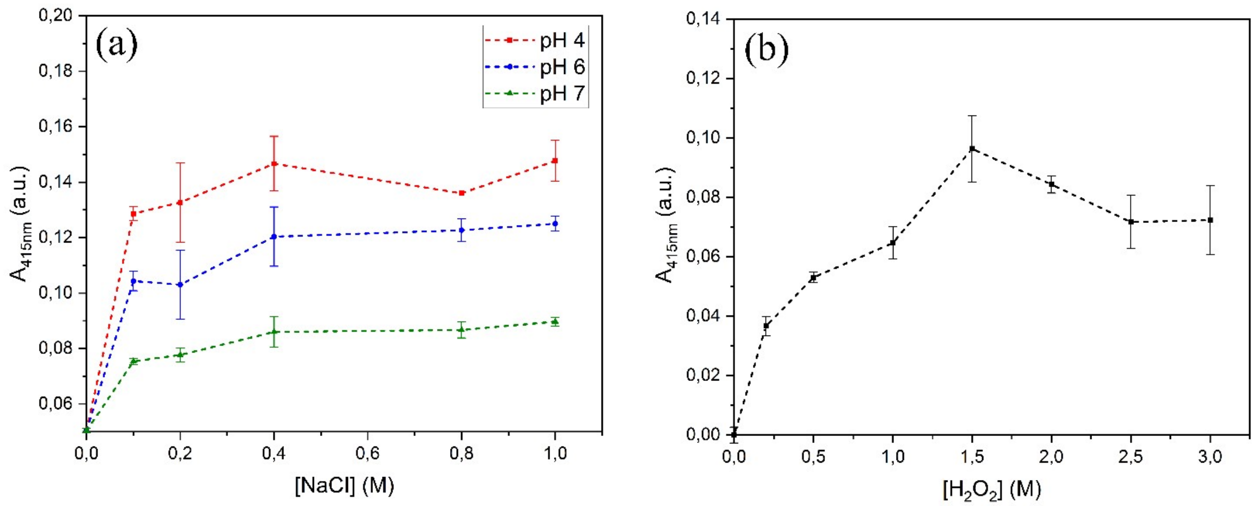

Fig. S9. Optimization of the enzymatic reaction conditions. a) Catalytic activity of AuNC under different pH conditions and with increasing NaCl concentration, b) Activity of the assay as a function of hydrogen peroxidase concentration.

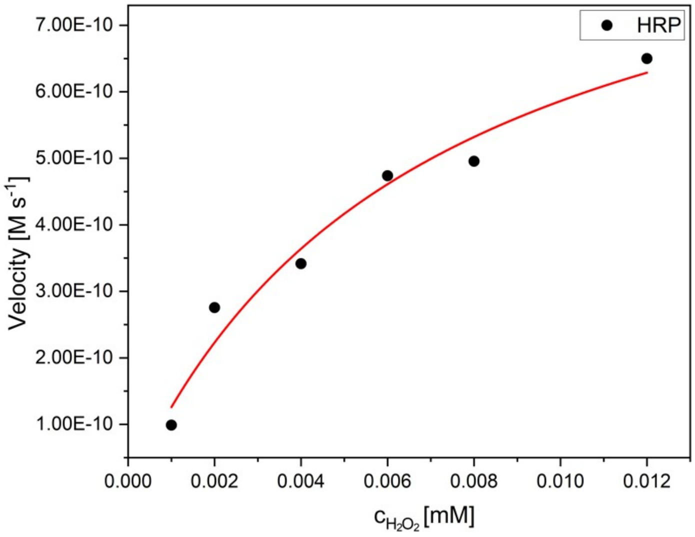

Fig. S10. Steady-state kinetic assay of HRP with low H_2O_2 concentration. Plot of velocity against H_2O_2 concentration with a constant ABTS concentration in which HRP was used as enzyme. The result reveals better affinity towards H_2O_2 in comparison to AuNC@Protein.

Table S2. Comparison of the kinetic parameters of HRP in the case of μM concentration range of H_2O_2 used as substrates at pH 4 and RT.

	c_{enzyme} [M]	Substrate	K_M [mM]	V_{max} [M s^{-1}]	K_{cat} [s^{-1}]
HRP	3.5 x 10^{-11}	H_2O_2	0.007	0.99 x 10^{-9}	0.28 x 10^{2}

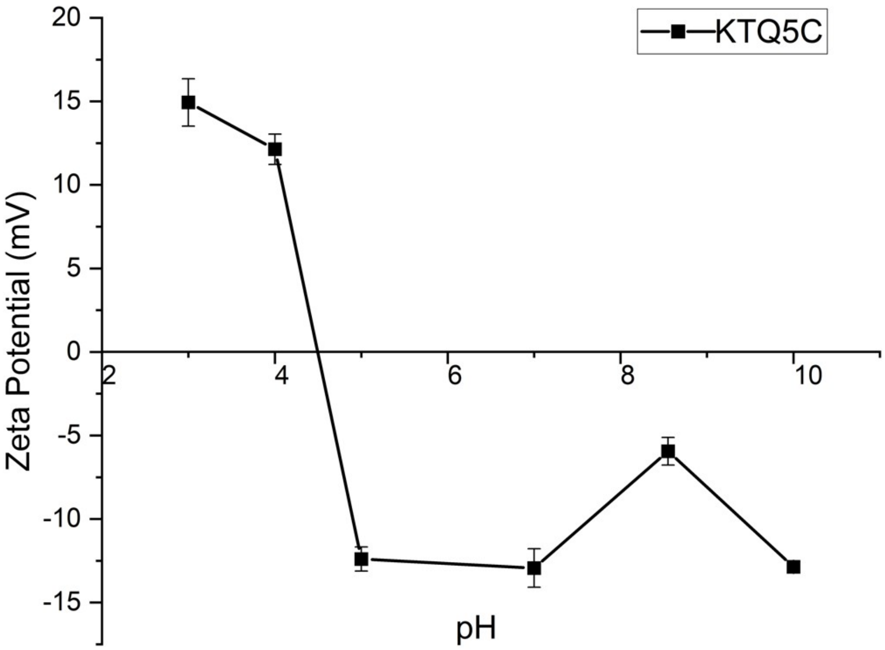

Fig. S11. ζ-potential graph of KTQ5C providing information related to the charge distribution at different pH values.

Table S3: Comparison of the proposed approach with other peroxidase-like nanozymes for H_2O_2 detection.

	Enzyme [M]	Substrate	K_M [mM]	V_{max} [M s^{-1}]	K_{cat} [s^{-1}]	Ref.
SH-NH_2-MION	N.R.	ABTS	0.25	1.7 x 10^{-7}	N.R.	5
SH-NH_2-MION	N.R.	H_2O_2	5.7	1.8 x 10^{-7}	N.R.	
PB-NPs	N.R.	ABTS	157.45	N.R.	N.R.	6
PB-NPs	N.R.	H_2O_2	0.028	N.R.	N.R.	
Fe_3O_4@Au–Cys–FA	N.R.	ABTS	0.36	2.03 x 10^{-8}	N.R.	7
Fe_3O_4@Au–Cys–FA	N.R.	H_2O_2	0.44	10.9 x 10^{-8}	N.R.	
Co/Fe-MSN	1.85	ABTS	0.072	8.50 x 10^{-8}	0.62	8
Co/Fe-MSN	1.85	H_2O_2	0.387	5.80 x 10^{-8}	81.2	
AuNC@KTQ5C	6.6 x 10^{-8}	ABTS	0.062	34.0 x 10^{-9}	0.52	This work
AuNC@KTQ5C	6.6 x 10^{-8}	H_2O_2	0.040	9.30 x 10^{-9}	0.14	

SH-NH_2-MION: SH-NH_2-modified magnetic iron oxide nanoparticles
PB-NPs: Prussian blue nanoparticles
Fe_3O_4@Au–Cys–FA: folic acid–cysteine-conjugated gold-coated magnetite nanoparticles
Co/Fe-MSN: Co/Fe-functionalized mesoporous silica nanoparticles
N.R.: not reported

Table S4: Catalytic reaction conditions used for the evaluation of Michaelis Menten kinetics.

	**Buffer**	**pH**	**Ionic strength [I]**	**T [°C]**	**Ref.**
SH-NH_2-MION	PBS	4.0	N.R.	RT	[5]
PB-NPs	Acetate buffer (0.2 M)	4.0	0.2	50	[6]
Fe_3O_4@Au–Cys–FA	Acetate buffer (0.2 M)	4.0	0.2	45	[7]
Co/Fe-MSN	Acetate buffer	4.0	N.R.	25	[8]
AuNC@KTQ5C	PBS	4.0	0.4	RT	This work

References

1 P. H. Brown, A. Balbo, H. Zhao, C. Ebel and P. Schuck, *PLoS One*, 2011, **6**, e26221-26237.

2 W.G. Martin, C.A. Winkler and W. H. Cook, *Can. J. Chem.*, 1959, **37**, 1662-1670.

3 S. J. Edelstein and H. K. Schachman, *Journal of Biological Chemistry*, 1967, **242**, 306-311.

4 J. Xie, Y. Zheng and J. Y. Ying, *J Am Chem Soc*, 2009, **131**, 888-889.

5 Y. Liu and F. Yu, *Nanotechnology*, 2011, **22**, 145704-145708.

6 W. Zhang, D. Ma and J. Du, *Talanta*, 2014, **120**, 362-367.

7 K. Ponlakhet, M. Amatatongchai, W. Sroysee, P. Jarujamrus and S. Chairam, *Analytical Methods*, 2016, **8**, 8288-8298.

8 M. Aghayan, A. Mahmoudi, M. R. Sazegar and F. Adhami, *J Mater Chem B*, 2021, **9**, 3716-3726.
